# A secreted splice variant of the Xenopus frizzled-4 receptor is a biphasic modulator of Wnt signalling

**DOI:** 10.1186/1478-811X-11-89

**Published:** 2013-11-19

**Authors:** Anne-Kathrin Gorny, Lilian T Kaufmann, Rajeeb K Swain, Herbert Steinbeisser

**Affiliations:** 1 Section Developmental Genetics, Institute of Human Genetics, University of Heidelberg, Im Neuenheimer Feld 366, Heidelberg D-69120, Germany; 2 Vascular Biology Laboratory, Institute of Life Sciences, Nalco Square, Bhubaneswar 751023, India

**Keywords:** Fz4-v1, Splice variant frizzled-4 receptor, Biphasic, Wnt signalling, SFRP, *Xenopus*, Dorsal fin, Neural crest

## Abstract

**Background:**

Activation of the Wnt signalling cascade is primarily based on the interplay between Wnt ligands, their receptors and extracellular modulators. One prominent family of extracellular modulators is represented by the SFRP (secreted Frizzled-related protein) family. These proteins have significant similarity to the extracellular domain of Frizzled receptors, suggesting that they bind Wnt ligands and inhibit signalling. The SFRP-type protein Fz4-v1, a splice variant of the Frizzled-4 receptor found in humans and *Xenopus,* was shown to augment Wnt/β-catenin signalling, and also interacts with those Wnt ligands that act on β-catenin-independent Wnt pathways.

**Findings:**

Here we show that *Xenopus* Fz4-v1 can activate and inhibit the β-catenin-dependent Wnt pathway. Gain-of-function experiments revealed that high Wnt/β-catenin activity is inhibited by low and high concentrations of Fz4-v1. In contrast, signals generated by low amounts of Wnt ligands were enhanced by low concentrations of Fz4-v1 but were repressed by high concentrations. This biphasic activity of Fz4-v1 was not observed in non-canonical Wnt signalling. Fz4-v1 enhanced β-catenin-independent Wnt signalling triggered by either low or high doses of Wnt11. Antisense morpholino-mediated knock-down experiments demonstrated that in early *Xenopus* embryos Fz4-v1 is required for the migration of cranial neural crest cells and for the development of the dorsal fin.

**Conclusions:**

For the first time, we show that a splice variant of the Frizzled-4 receptor modulates Wnt signalling in a dose-dependent, biphasic manner. These results also demonstrate that the cystein-rich domain (CRD), which is shared by Fz4-v1 and SFRPs, is sufficient for the biphasic activity of these secreted Wnt modulators.

## Findings

### Fz4-v1 demonstrates a dose-dependent, biphasic activity in modulating Wnt/β-catenin-dependent signalling

SFRPs consist of two domains, an N-terminal cysteine-rich domain (CRD) and a C-terminal Netrin-like domain [1-3]. Based on similarity with the CRD region of Frizzled receptors, SFRPs were originally described as classical Wnt antagonists [4], however, recent data also describe agonistic functions of SFRPs [5-9]. These gain-of-function experiments in cultured cells and *Xenopus* embryos demonstrated that low levels of SFRPs enhance the Wnt/β-catenin pathway, but high levels of SFRPs suppress Wnt signalling [7,8].

*Xenopus* Fz4-v1 is an SFRP-type protein which is generated from the Frizzled-4 receptor mRNA by intron retention. In contrast to classical SFRPs Fz4-v1 includes a CRD domain but lacks a Netrin-like domain [9]. Such splice variants have been described for both the human and *Xenopus* Frizzled-4 receptor and were previously named Fz4S [5,9]. *Xenopus* Fz4-v1 is a secreted protein and can modulate Wnt/β-catenin signalling in a non-cell autonomous manner (Additional file 1: Figure S1).

Both human and *Xenopus* Fz4 splice variants were shown to enhance the activity of Wnt ligands, which activate the Wnt/β-catenin pathway [5,9]. *Xenopus* Fz4-v1 also interacts with Wnt ligands of the Wnt5a class, but its effect on β-catenin-independent Wnt signalling has not been assessed [9]. Because previous experiments had only demonstrated an activating function of Fz4-v1, we tested whether Fz4-v1 might also have an inhibitory activity on Wnt/β-catenin signalling.

**Figure 1:**
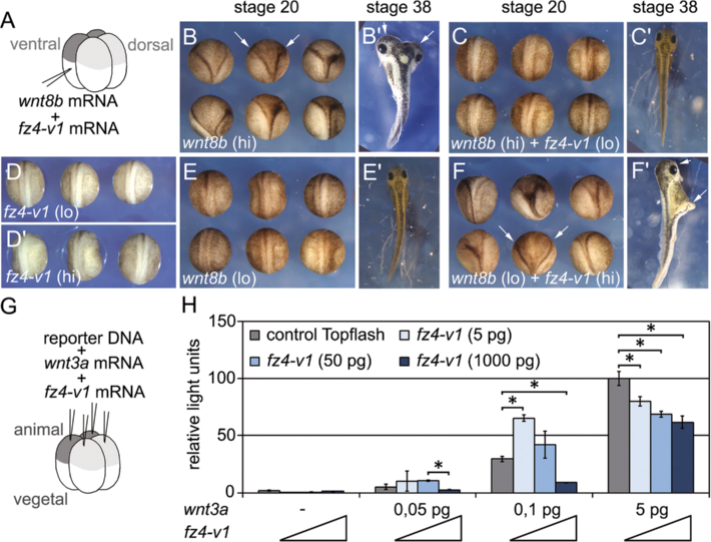
**Fz4-v1 has a biphasic, dose-dependent activity in modulating Wnt/β-catenin-dependent signalling. (A-F)** 4-cell stage embryos were injected on the ventral marginal side with 0.5 pg *wnt8b* RNA (lo) or 10 pg (hi) alone, or in combination with 250 pg *fz4-v1* RNA (lo) or 1000 pg (hi) or with 250 pg (lo) or 1000 pg (hi) *fz4-v1* RNA alone. Formation of secondary body axes was scored at neurula (st. 20, **B**-**F**) and tadpole stages (st. 38, **B’**-**F’**). Frequency of secondary axis formation: **(B, B’)** 71.9% (n = 36), **(C, C’)** 0% (n = 24), **(D, D’)** 0% (n = 20), **(E, E’)** 0% (n = 24), **(F, F’)** 54.2% (n = 24). **(G)** At the 4-cell stage blastomeres were injected in the animal region with 80 pg Topflash-Luciferase reporter plasmid in combination with *wnt3a* RNA (0.05 pg, 0.1 pg, 5 pg) and *fz4-v1* RNA (5 pg, 50 pg, 1000 pg). **(H)** Luciferase activity was measured at gastrula stage (st. 11). Error bars represent standard deviation (SD). (*) indicates significant difference (Student’s *t* test, p < 0.05).

To investigate the dual function of Fz4-v1, we took advantage of the *Xenopus* axis duplication assay, which provides a sensitive and reliable system to test Wnt activities (Figure 1A-F). Injection of 10 pg *wnt8b* RNA into the ventral marginal zone caused ectopic axis formation in more than 70% of the injected embryos. However, co-injection of 250 pg *fz4-v1* RNA completely blocked the formation of secondary body axes. When low doses of *wnt8b* (0.5 pg) were injected, which alone were not sufficient to induce a secondary axis, co-injection of 1 ng *fz4-v1* induced ectopic axes in more than 50% of the embryos, consistent with previous studies [5].

In addition, we performed Topflash-Luciferase reporter experiments in *Xenopus* embryos in order to quantitatively measure Wnt/β-catenin signalling strength (Figure 1G, H). *Xenopus* embryos were injected with synthetic mRNAs for *wnt3a*, *fz4-v1* and the Topflash-Luciferase reporter plasmid. Wnt activity was measured at gastrula stage. Fz4-v1 alone did not activate the Topflash-Luciferase reporter. However, Luciferase activity generated by low doses of *wnt3a* (0.05 pg and 0.1 pg) was enhanced by co-injection of 5 and 50 pg of *fz4-v1*, but repressed by 1000 pg. In contrast, strong Wnt/β-catenin signals triggered by high doses of *wnt3a* (5 pg) were inhibited by both, high (1000 pg) and low (5 and 50 pg) amounts of *fz4-v1* (Figure 1H).

This analysis revealed that Fz4-v1 can act as a biphasic modulator of Wnt/β-catenin signalling. Therefore Fz4-v1 behaves like classical SFRPs, which activate Wnt signalling at low and inhibit at high doses [7,8]. The presence of the CRD domain is sufficient to induce biphasic activity, because Fz4-v1 is lacking the NTR domain. Recent structural analysis of Wnt8 interaction with the CRD domain of Fz8 could explain the finding that low doses of SFRPs activate, but high doses inhibit the activity of Wnt ligands [10]. Low CRD concentration could weaken the attachment of the lipid modified Wnt proteins to the plasma membrane and ECM. Wnts would become more diffusible and association with the receptors would be facilitated. High concentration of CRD could induce the clustering of Wnt/CRD complexes, rendering them inactive. Since SFRPs can bind to Frizzled receptors, it would also be plausible that high concentrations of secreted CRD domains could cause receptor silencing.

In case of strong Wnt/β-catenin signals induced by high concentration of Wnt ligands one can assume that all the endogenous Wnt receptors are occupied. In this case the CRD domains could compete with Wnt ligands for the receptors, which would result in reduced Wnt/β-catenin activity.

### Fz4-v1 activates the Wnt/β-catenin-independent JNK pathway

**Figure 2:**
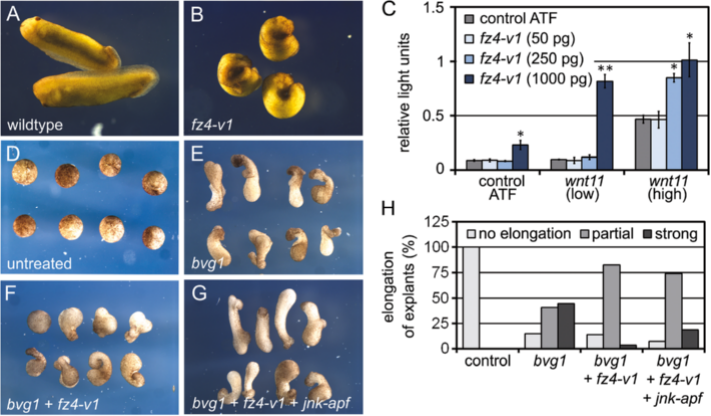
**Fz4-v1 perturbs morphogentic movements by activation of the Wnt/β-catenin-independent JNK pathway. (A, B)** Embryos were injected radially at the 4-cell stage with 1 ng *fz4-v1* RNA. At tailbud stages (st. 25) 22% displayed spina bifida and neural tube closure defects (n = 45). **(C)** At the 4-cell stage 50 pg of the ATF-luciferase reporter plasmid was injected ventral-animally alone, or in combination with 100 pg (low) or 500 pg (high) *wnt11* RNA and *fz4-v1* RNA (5, 50 and 1000 pg). Reporter activity was analyzed at late gastrula stages (st. 12). Error bars represent SD. (*) indicate significant difference to control or *wnt11* alone, respectively (grey bars) (Student’s *t* test, *p < 0.05, **p < 0.001). **(D-H)** 4-cell stage embryos were injected at the animal pole region with *bvg1* RNA (100 pg) alone, or in combination with *fz4-v1* (600 pg) and *jnk-apf* (1 ng). At blastula stages (st. 9) the caps were explanted and elongation was scored at stage 25. **(D)** Non-injected controls (n = 20) showed no elongation but *bvg1*-injected explants **(E)** did (n = 27). **(F)** Co-injection of *fz4-v1* partially inhibited explant elongation (n = 29), which was restored by JNK-APF (n = 27) **(G). (H)** Quantification of the animal cap experiments.

Wnt5a-type ligands such as Wnt5a and Wnt11 regulate β-catenin-independent signalling. In *Xenopus* embryos they orchestrate morphogenetic cell movements by regulating planar cell polarity (PCP) in the mesoderm and the neuroectoderm. Since Fz4-v1 can interact with Wnt5a and Wnt11, we hypothesized that it could interfere with non-canonical Wnt signalling [9]. Overexpression of Fz4-v1 in *Xenopus* embryos caused malformations such as shortened body axes and spina bifida, indicating that β-catenin-independent Wnt signalling was perturbed (Figure 2A, B). Since these phenotypes can be generated by either hyper-activation or inhibition of the PCP pathway, it was important to determine whether Fz4-v1 acts as activator or inhibitor.

PCP signalling can be measured using an ATF-Luciferase reporter [11], which we made use to analyze the effects of Fz4-v1 (Figure 2C). ATF reporter, *wnt11* and *fz4-v1* RNAs were injected animally into the two ventral blastomeres of *Xenopus* embryos at the 4-cell stage, and Luciferase activity was analyzed at gastrula stage. Low doses of *wnt11* were only able to induce ATF reporter activity when combined with high amounts of *fz4-v1*. ATF reporter activity induced by high concentrations of *wnt11* was further enhanced by co-injection of *fz4-v1* (Figure 2C). The ATF reporter experiment indicated that Fz4-v1 augments Wnt11-mediated PCP signalling. This was confirmed in *Xenopus* animal cap experiments (Figure 2D-G). Ectodermal animal cap explants stimulated by BVg1, a TGF-β growth factor [12], elongate due to convergent-extension (CE) movements that are controlled by the PCP pathway. Explants injected with RNA coding for BVg1 elongated as expected, but co-expression of Fz4-v1 inhibited CE movements. JNK is a downstream effector of the PCP pathway, and co-expression of a dominant negative form of *Xenopus* JNK1, JNK-APF, antagonized the inhibitory effect of Fz4-v1 in the explants (Figure 2D-G). This demonstrates that Fz4-v1 inhibits CE movements by hyper-activation of PCP signalling and confirms the results of the ATF reporter assay.

### In vivo function of Fz4-v1

To analyze the endogenous function of Fz4-v1 during *Xenopus* embryogenesis we performed antisense morpholino knock-down experiments using a translation-blocking morpholino that targets the 5′-UTR. The efficiency and specificity of the morpholino is shown in the Supplements (Additional file 2: Figure S2). Since Fz4-v1 is generated by intron retention, the antisense morpholino also blocks translation of *fz4* mRNA. Injection of the Fz4/Fz4-v1 morpholino at the 2-or 4-cell stage had no adverse effect on early embryogenesis, suggesting that Fz4-v1 and Fz4 do not affect maternal and early zygotic Wnt signalling, since such interference are expected to result in dorsoventral patterning defects.

**Figure 3:**
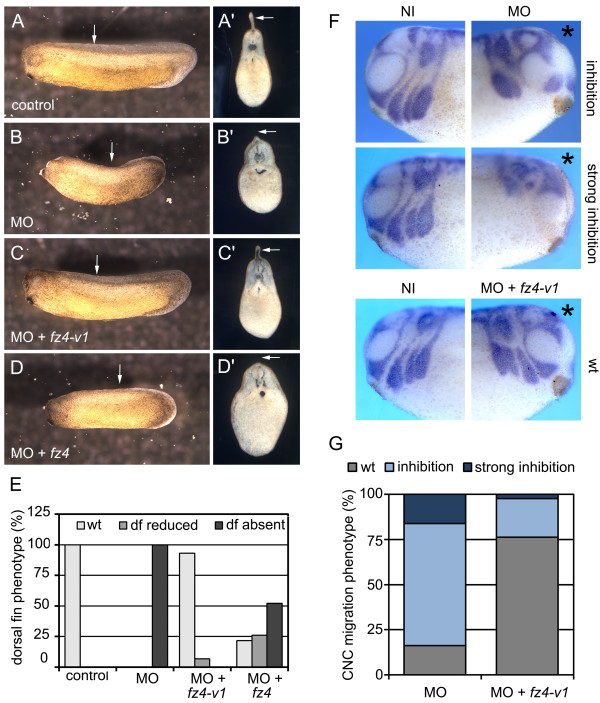
**Fz4-v1 function is required for dorsal fin development. (A-D)** Embryos were injected at the 2- to 4-cell stage with 50 ng antisense Fz4/Fz4-v1 morpholino oligonucleotide (MO) alone **(B, B’)** (n = 10), or in combination with 500 pg *fz4-v1* RNA **(C, C’)** (n = 29) or 500 pg *fz4* RNA **(D, D’)** (n = 23). At the tailbud stage (st. 30) formation of the dorsal fin was compared to uninjected control embryos **(A, A’)** (n = 40). **(A’-D’)** Vibratome cross-sections of the embryos shown in **A**-**D**. White arrows indicate the dorsal fin. **(E)** Quantification of the experiment. (df) dorsal fin. **(F)***in situ* hybridization for *sox10* and *twist* mRNA in tailbud-stage embryos (st. 32) injected unilaterally with Fz4/Fz4-v1 MO or the combination of MO and *fz4-v1* RNA. Asterices indicate the injected side of the embryos. (NI) non-injected side of the embryo, (wt) wild-type embryos. **(G)** Quantification of the CNC phenotypes.

At tailbud stages, however, Fz4/Fz4-v1-depleted embryos displayed defective dorsal fin development (Figure 3A-E). When the antisense morpholino was injected only in one side of the embryo *in situ* hybridization for *sox10* and *twist* mRNA revealed that migration of cranial neural crest (CNC) cells was inhibited (Figure 3F-G). Both, the dorsal fin phenotype and the CNC migration defect could be specifically rescued by co-injection of *fz4-v1* RNA. Since Fz4-v1 can rescue the morpholino effect despite its inability to generate a Wnt-dependent Fz4 signal, one can conclude that the morpholino phenotypes are not caused by the knock-down of the Fz4 receptor. Rescue experiments with the full length Fz4 receptor were much less effective (Figure 3D, D’), but Western blot analysis showed that the amount of Fz4-v1 protein was approximately four times higher than that of the full length Fz4 receptor (Additional file 2: Figure S2C). Increasing the amount of *fz4* four times to 2000 pg, however, resulted in severe malformations of the embryos (Additional file 2: Figure S2E, I). From these experiments we conclude that Fz4-v1 functions *in vivo* as a modulator of CNC migration and dorsal fin development. Classical experiments demonstrated a contribution of trunk neural crest cells to the mesenchyme in the core of the dorsal fin [13]. In addition, recent data also report an involvement of somites in dorsal fin development [14]. Notably, *fz4-v1* is expressed in the cranial and trunk neural crest, as well as the somitic mesoderm at neurula to tailbud stages (Additional file 2: Figure S2M-O), supporting an involvement of Fz4-v1 in dorsal fin induction and CNC migration. Neural crest, somites and dorsal fin formation are regulated by Wnt signalling and knock-down of the Wnt co-receptor Lrp6 in *Xenopus* embryos leads to reduced dorsal fins, similar to Fz4-v1-depleted embryos [15].

Furthermore, it was shown that a calcium-sensitive epithelial-mesenchymal-transition (EMT) event essential for dorsal fin induction is controlled by Wnt11-R [14], which could be modulated by Fz4-v1. Our data suggest that Fz4-v1 controls Wnt signalling in the head and trunk neural crest and somites, and thereby contributes to the development of the dorsal fin.

## Materials and methods

### Xenopus embryo manipulations

*Xenopus laevis* frogs were obtained from Nasco and all experiments complied with local and international guidelines for the use of experimental animals*. Xenopus* eggs were obtained from females injected with 500 IU human chorionic gonadotropin (Sigma), and were fertilized *in vitro*. Embryos were dejellied with 2% cysteine hydrochloride (pH8) and embryos were microinjected in 1x MBSH (88 mM NaCl, 1 mM KCl, 2.4 mM NaHCO_3_, 0.82 mM MgSO_4_, 0.41 mM CaCl_2_, 0.33 mM Ca(NO_3_)_2_, 10 mM HEPES (pH7.4), 10 μg/ml penicillin). The embryos were cultured in 0.1x MBSH and staged according to Nieuwkoop and Faber [16].

### Animal cap elongation assay

For the animal cap elongation assay, 4-cell-stage embryos were injected animally into two opposing blastomeres with synthetic RNAs. Animal caps were excised at stage 9 and cultivated in 1x MBSH together with 10 ng/μl gentamycine overnight.

### Synthesis of CAP-RNA and morpholinos for microinjection

Capped RNAs were synthesized form linearized plasmids using the mMessage mMachine Kit (Ambion).

pCS2-Wnt3a (mouse), pCS2-Wnt8b, pCS2-Wnt11, pCS2-Fz4, pCS2-Fz4-v1, pCS2-Fz4-myc, pCS2-Fz4-v1-myc and pCS2-JNK-APF (all *Xenopus*) were linearized with Not1 and pSP64T-BVg1 (*Xenopus*) was linearized with EcoRI. Sense RNA was transcribed by SP6 polymerase.

For knock-down experiments antisense Fz4/Fz4-v1 morpholino oligonucleotide (5′-ATTATTCTTCTTCTGTTGCCGCTGA-3′) or control morpholino (5′-CCTCTTACCTCAGTTACAATTTATA-3′) was injected.

### Whole-mount in situ hybridization and Luciferase reporter assay

Embryos were fixed in MEMFA and whole-mount *in situ* hybridization was performed as described [17]. pBluescriptSK–Sox10 [14] was linearized with EcoRI, and DIG-labelled antisense RNA was transcribed by T3 polymerase. pCR2.1–Twist [18] was linearized with HindIII, and DIG-labelled antisense RNA was transcribed by T7 polymerase. pCR-Blunt II-TOPO-Fz4-IntronI [9] was linearized with BamHI, and DIG-labelled antisense RNA was transcribed by T7 polymerase. Whole-mount *in situ* hybridization for *fz4-v1* was performed using a double (5′ and 3′) DIG-labelled LNA probe (5′-AGTATAGAAAGTAAACCCCCTGTG-3′) from Exiqon, according to manufacturer’s instructions.

For reporter assays 4-cell stage embryos were injected animally with 80 pg M50 Super 8x Topflash [19] or 50 pg ATF-Luciferase reporter plasmid [11] in combination with 8 pg or 5 pg TK-Renilla-Luciferase reporter plasmid. The reporter plasmids were injected alone or in combination with synthetic RNAs. Triplicates of 5 embryos were lysed according to the manufacturer’s protocol (Promega) and 20 μl of cell lysate was used for Luciferase detection.

### Additional information

Materials and Methods used for experiments in Additional files 1 and 2: Figures S1 and S2 are provided separately in Additional file 3.

## Competing interests

The authors declare that they have no competing interests.

## Authors’ contributions

AKG and HS designed the experiments. AKG, LTK and RKS performed the experiments and contributed to the writing of the manuscript. HS supervised the work and wrote the manuscript. All authors read and approved the final manuscript.

## Supplementary Material

Additional file 1: Figure S1 Fz4-v1 is a secreted protein during *Xenopus* development. Click here for file

Additional file 2: Figure S2 Specificity of Fz4/Fz4-v1 morpholino oligonucleotide mediated knock-down of Fz4 and Fz4-v1. Click here for file

Additional file 3 **Materials and methods **[20]**.** Click here for file
