# Supplementary material for: A secreted splice variant of the Xenopus frizzled-4 receptor is a biphasic modulator of Wnt signalling
Source: Cell Commun Signal. 2013 Nov 19;11:89. doi: 10.1186/1478-811X-11-89 (PMC4077065; doi:10.1186/1478-811X-11-89)
Supplement: Additional file 1: Figure S1 — Fz4-v1 is a secreted protein during Xenopus development. [file 1478-811X-11-89-S1.doc]

**Additional file 1: Figure S1.**

**
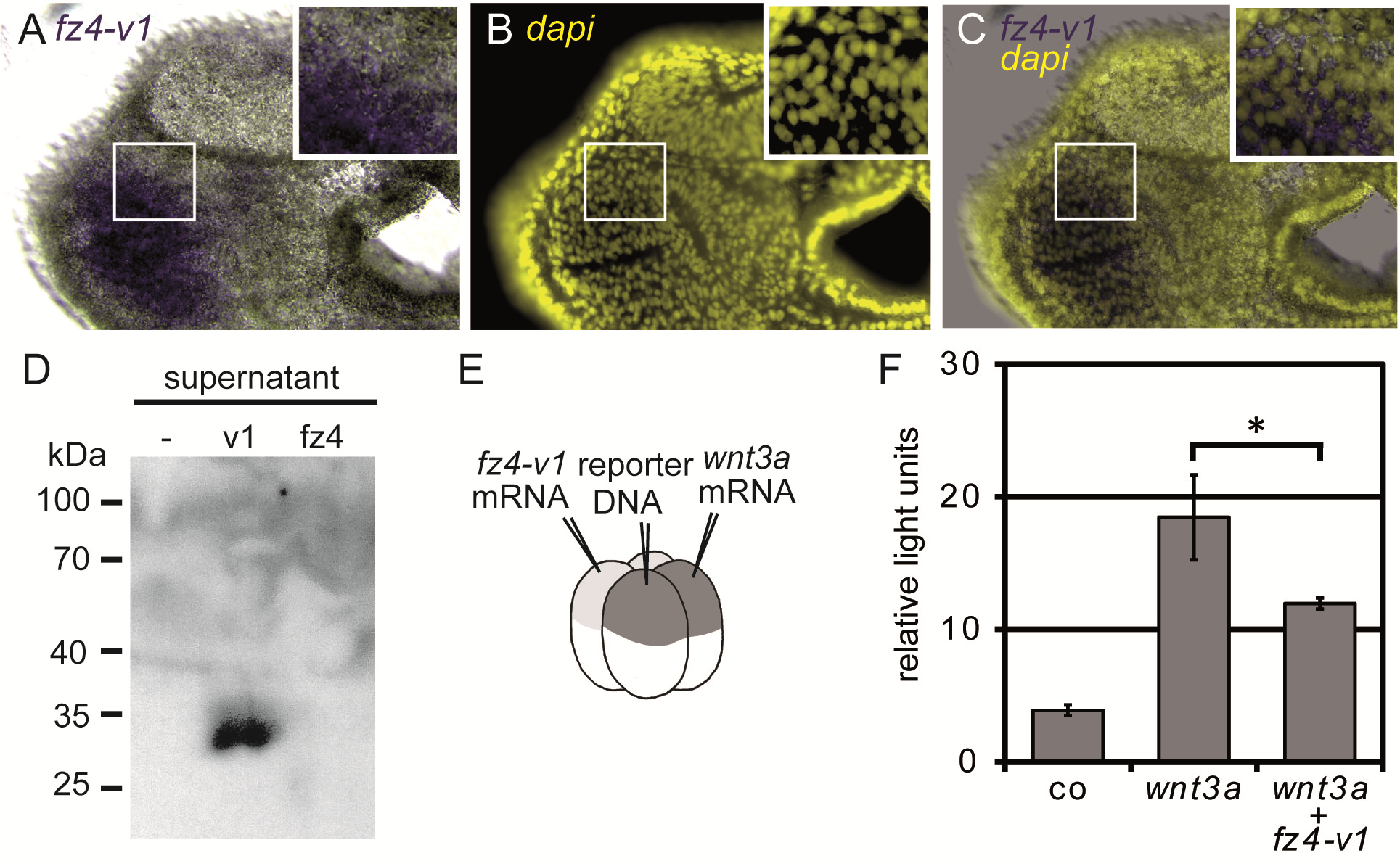
**

**Fz4-v1 is a secreted protein during *Xenopus* development.**

**(A-C)** Transversal sections of the head region of a wild type *Xenopus* tailbud embryo showing expression of *fz4-v1* by *in situ* hybridization with a probe targeting the retained intron (**A**, purple). Nuclei stained by DAPI (**B**, yellow). Overlay (**C**) shows that *fz4-v1* mRNA is present in the cytoplasm and not only in the nucleus, indicating that this is mature, translated *fz4-v1* mRNA. Insets: enlarged boxed areas.

(**D**) Western blot analysis for Fz4-v1-myc (~30kDa), and Fz4-myc (~90kDa) in the medium of untransfected (-), pCS2-Fz4-v1-myc (v1) or pCS2-Fz4-myc (fz4) transfected HEK293T cells, showing that Fz4-v1-myc protein, but not Fz4-myc, is secreted in HEK293T cells.

(**E-F**) At the 4-cell stage one blastomere was injected with 80 pg Topflash-Luciferase reporter plasmid. One adjacent blastomere was injected with 10 pg *wnt3a* RNA another with 250 pg of *fz4-v1* RNA as shown in the schematic drawing (**E**). The results of the Topflash reporter assays show that non-cell-autonomous activation of reporter by secreted Wnt3a, is inhibited by injecting *fz4-v1* RNA in a different blastomere (**F**, Error bars represent SD. (*****) indicates significant reduction (Student’s t test, p < 0.05)). This inhibition is also non-cell-autonomous and therefore requires the secretion of the Fz4-v1 protein.
