# Supplementary material for: A secreted splice variant of the Xenopus frizzled-4 receptor is a biphasic modulator of Wnt signalling
Source: Cell Commun Signal. 2013 Nov 19;11:89. doi: 10.1186/1478-811X-11-89 (PMC4077065; doi:10.1186/1478-811X-11-89)
Supplement: Additional file 2: Figure S2 — Specificity of Fz4/Fz4-v1 morpholino oligonucleotide mediated knock-down of Fz4 and Fz4-v1. [file 1478-811X-11-89-S2.doc]

**Additional file 2: Figure S2.**

**
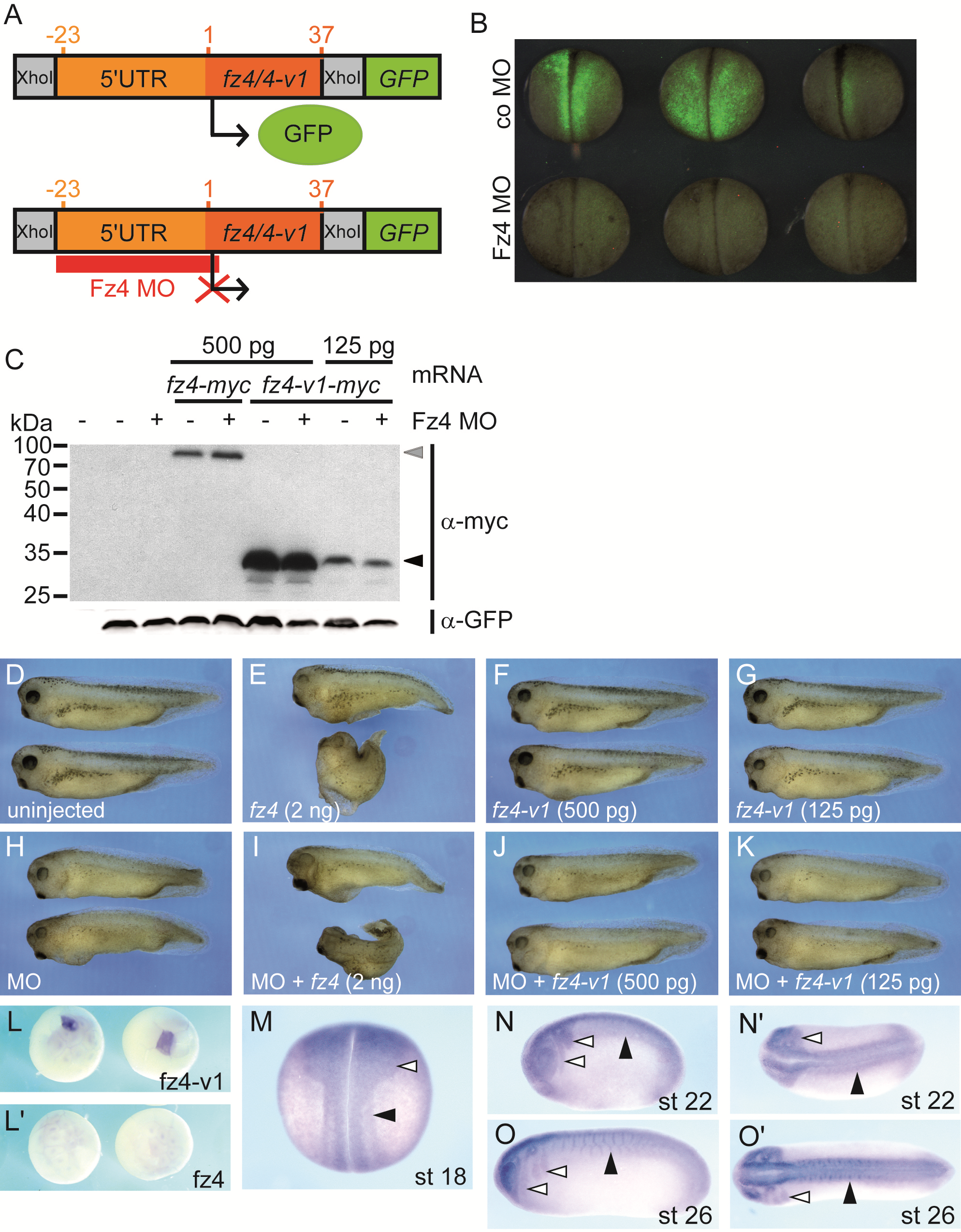
**

**Specificity of Fz4/Fz4-v1 morpholino oligonucleotide mediated knock down of Fz4 and Fz4-v1.**

(**A-B**) The Fz4/Fz4-v1 MO (Fz4 MO) targets the 5’UTR and ATG of *fz4* and *fz4-v1* mRNA. Embryos were co-injected equatorially at the 2-cell stage with 100 pg of a pCS2 construct containing the 5’UTR and start sequence of *fz4/fz4-v1* upstream of a GFP ORF (**A**, scheme) and with 50 ng of control MO (co MO) or Fz4 MO. Expression of GFP was observed in 95% (n=20) of the control embryos, while Fz4 MO (n=21) completely blocked translation of the construct (**B**).

(**C-K**) Embryos were co-injected at the 2-4 cell stage with 50 ng Fz4 MO and 100 pg *GFP* RNA alone or in combination with the indicated amounts of *fz4-myc* or *fz4-v1-myc* RNA. **(C)** At stage 20 five embryos of each sample were harvested to analyze the protein expression of Fz4-myc and Fz4-v1-myc. GFP was used to show that equal amounts of RNA were injected. Note that due to the larger size of *fz4-myc* RNA, similar amounts of injected *fz4-myc* and *fz4-v1-myc* RNA, led to 4 times less Fz4-myc protein compared to Fz4-v1-myc protein. The blot also shows the specificity of Fz4 MO, which does not block the translation of these *fz4/fz4-v1* RNAs, which lack the 5’UTR of *fz4/fz4-v1*. (**D-K**) Phenotype analyses of the injected embryos. To adjust the protein levels of Fz4-v1and Fz4, we used 4 times higher amounts (2000 pg) of *fz4-myc* RNA, and observed that this caused strong axis defects in 100 % of the embryos. (**E**, n=16), while *fz4-v1-myc* RNA alone caused no obvious phenotypes at both high (**F**, n=24) and 4 times lower doses (**G**, n=19). The dorsal fin defect (59.2 % absent df) of Fz4 MO injection (**H**, n=26) was not rescued by co-injection of *fz4-myc* RNA, and the strong axis defects caused by Fz4 overexpression remained dominant (**I**, n=24). Co-injection of high (**J**, n=20) and low (**K**, n=23) doses of *fz4-v1-myc* RNA, however, partially rescued the dorsal fin phenotype (12.5 % and 13.3 % absent df, respectively).

(**L-O**) Whole mount *in situ* hybridization for *fz4-v1* mRNA in neurula to early tailbud embryos. The DIG-labeled LNA probes, targeting the C-terminal end of the *fz4-v1* ORF, specifically detects injected *fz4-v1* RNA (**L**, 600 pg in one blastomere at the 16-cell stage), but not 600 pg of injected *fz4* RNA (**L’**).The detected expression of *fz4-v1* in stage 18 neurulea (**M**) and in stage 22 (**N, N’**) and stage 26 (**O, O’**) tailbuds in the cranial (white arrow heads) and trunk neural crest (black arrow head) underlines its potential function during CNC migration and dorsal fin development. (**L, L’**) animal view, (**B, M, N’, O’**) dorsal view, (**D-K, N, O**) lateral view.
