# Supplementary material for: A secreted splice variant of the Xenopus frizzled-4 receptor is a biphasic modulator of Wnt signalling
Source: Cell Commun Signal. 2013 Nov 19;11:89. doi: 10.1186/1478-811X-11-89 (PMC4077065; doi:10.1186/1478-811X-11-89)
Supplement: Additional file 3 — Materials and methods [20]. [file 1478-811X-11-89-S3.doc]

**Additional file 3 Materials and methods**

**Paraffin Sections**

For histology, previously *in situ* hybridized *Xenopus* embryos were fixed, embedded and sectioned as previously described [20]

**Protein expression analyses**

*Xenopus* embryos were injected with indicated amounts of myc-tagged *fz4-v1* or *fz4* RNA at 2-4-cell stage. The embryos were grown till late neurula stage (st. 20) and protein was extracted in 10 µl NP-40 lysis buffer as described [9].

HEK293T cells (2.5x10^5^) were seeded and cultivated in 6-well plates with 2 ml DMEM ReadyMix (Paa Laboratories) at 37°C and 5% CO_2_. After 24 h cells were transfected with 4 µg/well plasmid-DNA (pCS2-GFP, pCS2-Fz4-myc or pCS2-Fz4-v1-myc) using TurboFect Transfection Reagent (Thermo Scientific) according to manufacturer’s instruction. Transfection efficiency was confirmed by GFP fluorescence (>70 %). After 48 h the medium was harvested and centrifuged at 1000 rpm for 10 min. The cell-free supernatant was concentrated from 2 ml to 200 µl by centrifuging 40 min at 4000 g in Amicon® Ultra-4 Centrifugal Filter Device (3K) according to manufacturer’s instructions.

12.5 µl of the concentrated HEK293T supernatants were separated on a 12.5 % SDS-PAGE and proteins were detected by western blot analysis as described [9]. The antibodies used were monoclonal mouse anti-myc (9E10) and polyclonal rabbit anti-GFP (Abcam, ab290) and the respective peroxidase-conjugated secondary antibodies (Dianova).
